# Amyloid precursor protein-fragments-containing inclusions in cardiomyocytes with basophilic degeneration and its association with cerebral amyloid angiopathy and myocardial fibrosis

**DOI:** 10.1038/s41598-018-34808-7

**Published:** 2018-11-09

**Authors:** Lara Maria Krämer, Johannes Brettschneider, Jochen K. Lennerz, Daniel Walcher, Lubin Fang, Angela Rosenbohm, Karthikeyan Balakrishnan, Julian Benckendorff, Peter Möller, Steffen Just, Michael Willem, Albert C. Ludolph, Dietmar Rudolf Thal

**Affiliations:** 1 Institute of Pathology, Ulm University, Ulm, Germany; 2 RehaClinic Lucerne, Lucerne, Switzerland; 3 Department of Pathology, Massachusetts General Hospital/Harvard Medical School, Boston, MA USA; 4 Department of Internal Medicine II (Cardiology), Ulm University, Ulm, Germany; 5 Department of Neurology, Ulm University, Ulm, Germany; 6 Department of Gene Therapy, Ulm University, Ulm, Germany; 7 Biomedical Center, Ludwig-Maximilians-Universität Munich, Munich, Germany; 8 Department of Neuroscience and Leuven Brain Institute, KU-Leuven, Leuven, Belgium; 9 Department of Pathology, UZ-Leuven, Leuven, Belgium

## Abstract

Cardiomyopathies with intracellular inclusions are a distinct subset of cardiomyopathies whereas basophilic degeneration (BD) of the heart describes inclusions in cardiomyocytes of the aging heart, which have not yet been related to a specific disease condition or to a distinct type of protein inclusion. To address the question whether BD represents a specific pathological feature and whether it is linked to a distinct disease condition we studied 62 autopsy cases. BD inclusions exhibited an immunohistochemical staining pattern related to glycosylated, δ- or η-secretase-derived N-terminal cleavage products of the amyloid precursor protein (sAPPδ/η) or shorter fragments of sAPPη. BD aggregates were found in the myocardium of both ventricles and atria with highest amounts in the atria and lowest in the interventricular septum. The frequency of BD-lesions correlated with age, degree of myocardial fibrosis in individuals with arterial hypertension, and the severity of cerebral amyloid angiopathy (CAA). The intracytoplasmic deposition of N-terminal sAPPδ/η fragments in BD indicates a specific inclusion body pathology related to APP metabolism. The correlation with the severity of CAA, which is related to the APP-derived amyloid β-protein, supports this point of view and suggests a possible link between myocardial and cerebrovascular APP-related lesions.

## Introduction

Cardiomyopathies with intracellular inclusions are a distinct subset of cardiomyopathies. Major forms are the desmin-related myopathies in skeletal and heart muscle^1^ or the myocardial affection in inclusion body myopathy with early-onset Paget disease with or without frontotemporal dementia (IBMPFD)^2^. In addition to disease-related cardiomyocyte inclusions basophilic degeneration of the heart (BD) represents age-related basophilic inclusions in cardiac myocytes^3,4^. The inclusions were originally described based on hematoxylin and eosin (H&E)-stained sections as “a small, round, oval, or irregular pale blue area inside of a single muscle cell”^4^. These lesions are positive in the periodic acid-Schiff reaction staining (PAS)^5,6^ and polyglucosan immunohistochemistry^7^ raising the idea that BD consists of glycogen metabolism by-products^5,7^. At the electron microscopic level BD consists of randomly distributed fibrils rimmed by myofibrils^5^. However, a distinct pathological role of BD has not yet been identified.

**Figure 1:**
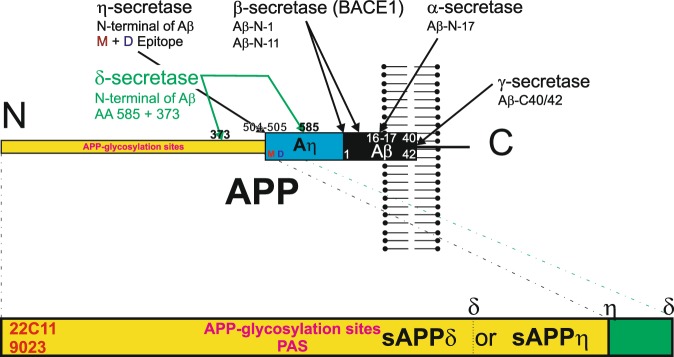
Schematic representation of APP cleavage by α-, β-, γ-, δ- and η-secretase and generation of sAPPδ and sAPPη. The δ-secretase can thereby cut at the amino acid position 373 as well as at position 585. As such, δ-secretase cleavage can produce a longer N-terminal fragment that is detectable with antibodies against the D- and M-epitope sAPPδ_585_ and fragments that do not contain these epitopes, i.e. sAPPδ_373_^17^. sAPPδ_373_ and sAPPη can be detected with antibodies against the N-terminus of APP (22C11, 9023) but not with antibodies detecting APP C-terminal to the δ_373_ or η-secretase cleavage site. sAPPδ/η contain glycosylation sites possibly explaining the detection of its glycosylated form stained by the PAS-method. The antibodies used for APP-staining do not differentiate between sAPPδ_373_, sAPPη and shorter N-terminal fragments of APP.

The amyloid precursor protein (APP) is known as precursor protein for the Alzheimer’s disease (AD)-related amyloid β-protein (Aβ)^8^. Aβ is released after β- and γ-secretase cleavage^9^ and is considered to be a key protein in the pathogenesis of AD^10–12^. The deposition of Aβ in the wall of leptomeningeal and cerebral blood vessels is the hallmark lesion of cerebral amyloid angiopathy (CAA)^13^, which develops not only linked to AD-related Aβ-production as shown in APP-transgenic mice^14^ but also in spontaneous hypertensive, stroke-prone rats (SHRSP)^15,16^ in the absence of amyloid plaques indicating a potential association between CAA and arterial hypertension. Moreover, δ- and η-secretase cleavage has been described cutting APP N-terminal to the β-secretase cleavage site releasing C-terminal fragments called CTFδ (by δ-secretase cleavage) and CTFη (by η-secretase cleavage) and N-terminal fragments called sAPPδ and sAPPη^17,18^. Aη is generated in a second cleavage step cutting CTFη by α- or β-secretase into Aη-α/β and CTFα/β^18^ (Fig. 1). APP-like proteins (APLPs) are homologues of APP lacking the Aβ region and are termed APLP1 and APLP2^19–21^. The role of APP, its cleavage products or its homologues APLP1 or APLP2 in heart pathology is not fully understood although it is known that APP and APLP2 are expressed in cardiomyocytes^19^ and some authors found that Aβ may play a role for cardiomyocyte degeneration in patients with heart failure^22^. In myofibrillar myopathy of the skeletal muscle accumulation of APP-positive material has been reported^23^. In so doing, the question arises whether Aβ/APP metabolism plays a role for the development of cardiac pathological lesions and if so whether they are related to AD or CAA.

To address this question and to clarify the role of BD as an age-related lesion of the heart we studied 62 autopsy cases. Our results showed that BD-lesions consist of N-terminal APP-fragments and were associated with the severity of CAA and with myocardial fibrosis in individuals with arterial hypertension.

## Results

### sAPPδ_373_/η epitopes are exhibited in p62/SQSTM1-, ubiquitin-, and PAS-positive BD-inclusions of cardiomyocytes

**Figure 2:**
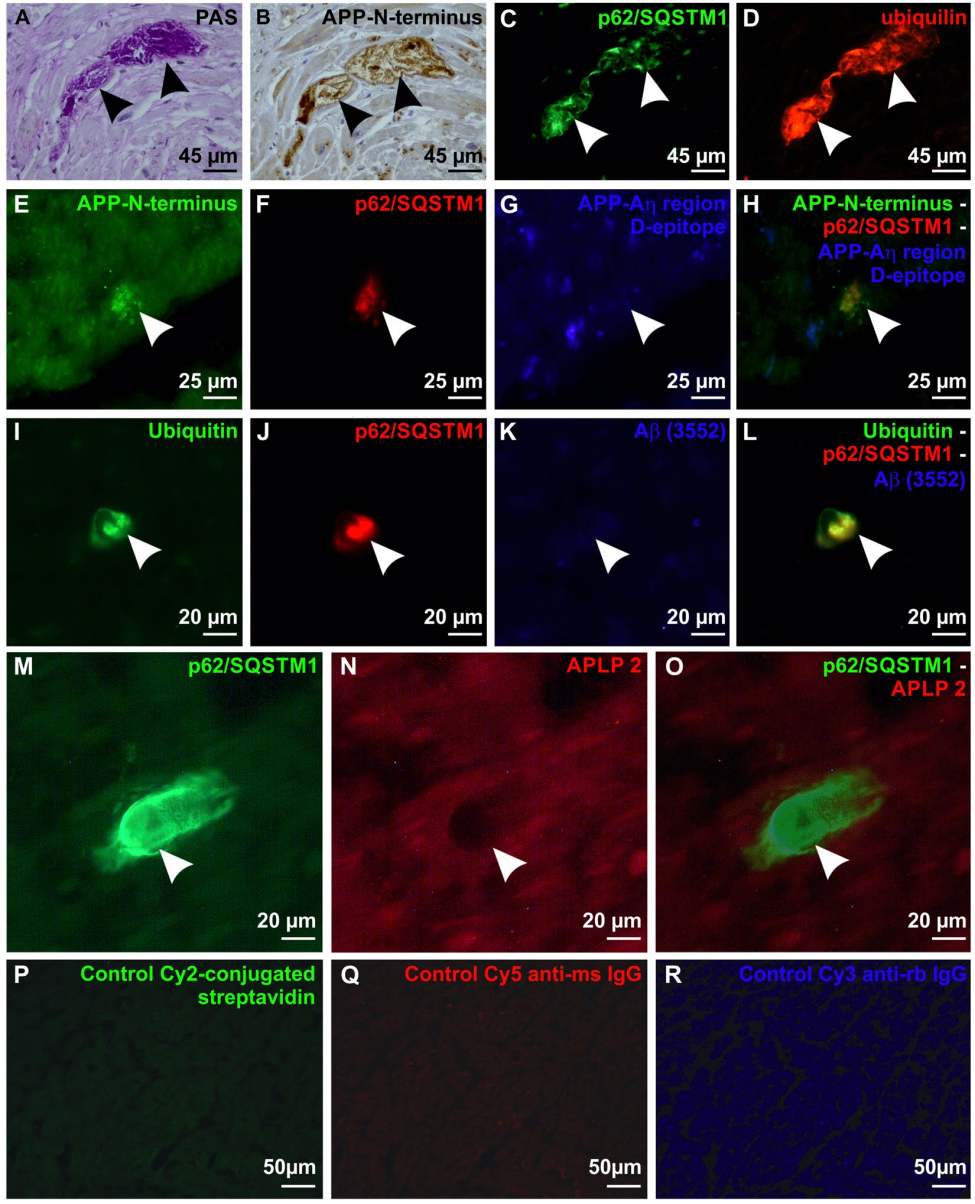
Ubiquitinated and glycosylated p62/SQSTM1-immunopositive BD-inclusions exhibiting N-terminal epitopes of APP in cardiomyocytes in cross sections through the left ventricle wall. (**A**) BD-inclusion (arrows) stained with the PAS histochemical staining method in a cardiomyocyte of the left ventricular myocardium in case No. 53. (**B**) Subsequent section of the BD-inclusion of case No. 53 depicted in **A** immunostained with anti-APP (22C11) antibodies against the N-terminus of APP (arrows). Note that neighboring cardiomyocytes exhibit a physiological APP expression in the cytoplasm, focally associated with lipofuscin granules. (**C**) p62/SQSTM1- immunohistochemistry showed in another subsequent section of the BD-inclusion of case No. 53 depicted in (**A**,**B)** the presence of p62/SQSTM1 (arrows). The aggregates exhibited an amorphous structure and were sharply delineated. (**D)** The section shown in **C** was also stained for ubiquilin by double label immunofluorescence, which was colocalizing with p62/SQSTM1 in the BD-lesion (arrows). (**E**–**H**) Triple label immunofluorescence of a BD-lesion in the myocardium of the left ventricle of case No. 17. The BD-lesion was stained with anti-APP (22C11) detecting the N-terminus of APP (arrow in **E**,**H**) and p62/SQSTM1 (arrow in **F**,**H**) whereas the antibody against the D-epitope of the Aη-region did not detect this lesion (arrow in **G**,**H**). (**I–L**) Triple label immunofluorescence of a BD-lesion in case No. 50 shows expression of ubiquitin (arrow in **I**,**L**) and p62/SQSTM1 (arrow in **J**,**L**) but no expression of Aβ detected with the antibody (3552)^44^ (arrow in **K**,**L**). (**M–O)** Double label immunofluorescence of a BD-lesion in the left ventricular myocardium of case No. 56 showed a p62/SQSTM1-positive BD inclusion (arrowhead in **M**,**O**) which was not visible with the antibody against APLP2 (arrowhead in **N**,**O**) whereas the cardiomyocytes showed a mild physiological cytoplasmic staining of APLP2. (**P–R**) Negative controls (myocardium of the left ventricle from case No. 17) for immunofluorescence stainings were performed by omitting primary antibodies (only secondary antibodies/detection agents): Carbocyanin-2 (Cy2)-conjugated streptavidin (**P**) Cy5-conjugated anti-mouse IgG (**Q**) and Cy3-conjugated anti-rabbit IgG secondary antibodies (**R**). Positive controls are depicted in Suppl. Fig. 1.

**Figure 3:**
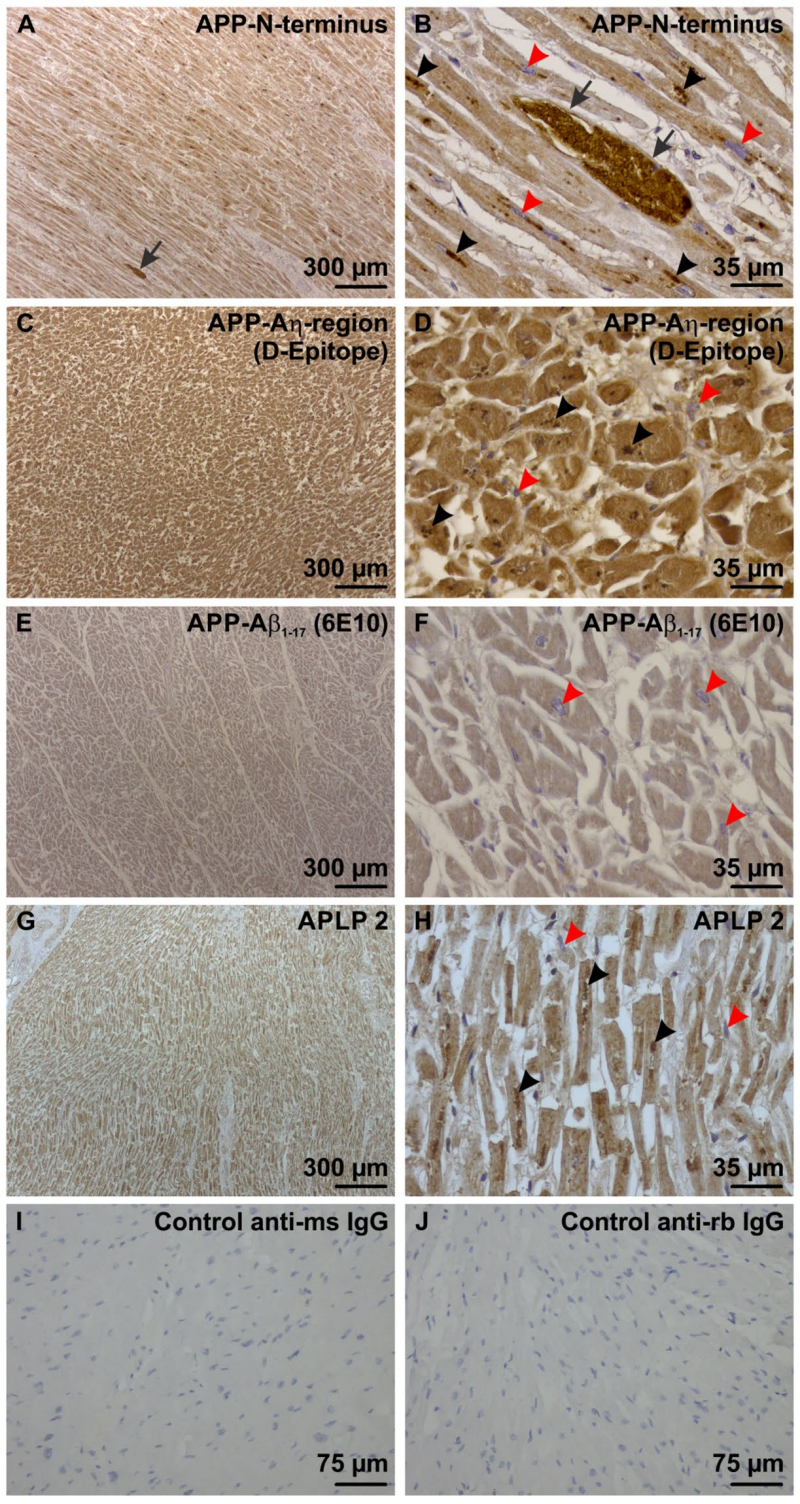
Physiological expression of APP and APLP2 in heart muscle cells of the left ventricle. (**A**) With an antibody directed against the N-terminus of APP there was a mild cytoplasmic staining in all cardiomyocytes of a left ventricle sample from case No. 15 exhibiting longitudinal-skewed cut muscle fibers. One BD lesion was seen (arrow) (**B**) In these cells lipofuscin particles (black arrowheads) were also stained as seen at the higher magnification level. The BD-inclusion (arrow) was morphologically different from the lipofuscin particles because it was bigger and showed a homogenous mass of strongly APP-positive material in the mildly APP expressing muscle cells. The nuclei of the cardiomyocytes were not stained and serve as intrinsic negative control for this staining (red arrowheads). (**C**) The Aη-D-epitope of APP was also expressed in all cardiomyocytes, here shown in a sample of the left ventricle myocardium of case No. 15. The muscle fibers were cut transversally. (**D**) At higher magnification level lipofuscin particles were also stained with the antibody against the Aη-D-Epitope (black arrowheads). BD-lesions were not seen with this antibody. The nuclei of the cardiomyocytes were not stained and served as intrinsic negative control for this staining (red arrowheads). (**E**,**F)** The Aβ epitope of APP was only faintly stained in cardiomyocytes of the left ventricle myocardium of case No. 58 using an antibody raised against Aβ_1–17_. No BD-lesions could be seen. Lipofuscin was not stained as well. The nuclei of the cardiomyocytes were not stained and served as intrinsic negative control for this staining (red arrowheads). The muscle fibers were cut transversally. (**G**) APLP2 was expressed in the cytoplasm of all cardiomyocytes of the left ventricular myocardium of case No. 1 exhibiting muscle fibers in longitudinal-skewed orientation. (**H**) At the higher magnification level lipofuscin particles (black arrowheads) were stained with this antibody as well. BD-lesions could not be detected with this antibody. The nuclei of the cardiomyocytes were not stained and served as intrinsic negative control for this staining (red arrowheads). (**I**,**J**) Negative controls by omitting the primary antibodies for biotinylated anti-mouse IgG (**I**) and anti-rabbit IgG secondary antibodies (**J**) were performed on left ventricular myocardium of case No. 44. Positive controls are shown in Suppl. Fig. 1.

To clarify the nature of BD-lesions we stained cross sections through the myocardium of the left ventricle with H&E, antibodies against p62/SQSTM1 and with the PAS-staining from all 62 cases included in this study (Table 1, Suppl. Table 1). PAS-positive BD-lesions in cardiomyocytes were found to express p62/SQSTM1 as shown in subsequent sections (Fig. 2A,C,F,H,J,L,M,O). To clarify whether other markers for an impaired proteasomal degradation of proteins confirm this finding and to screen for proteins that could be subject of impaired proteasomal degradation we screened cases No. 1, 15, 17, 50, 53, 56 and 58 (see Suppl. Table 1) with the antibodies listed in Suppl. Table 2. The pattern seen for BD-lesions was reproducible in all seven investigated cases. Antibodies against ubiquitin and ubiquilin were used to confirm an impaired proteasomal degradation, antibodies against abnormal τ-protein, α-synuclein, phosphorylated transactive response DNA-binding protein pTDP43, and Aβ/APP to identify potential target proteins that can theoretically accumulate in the cell, and antibodies against muscle-derived proteins to address the question whether BD-lesions represent a disorder of the cardiomuscular intermediary filaments. The p62/SQSTM1-positive BD-lesions were immunopositive for ubiquitin, ubiquilin, and N-terminal epitopes of APP (Fig. 2B,D,E,H,I, Suppl. Table 2). The M- and D-epitope representing the Aη-region of APP as well as the Aβ fragment were not detected (Fig. 2G,H,K,L, Table 1). An antibody directed against APLP2 did not stain the p62/SQSTM1-positive BD inclusions as demonstrated by double label immunohistochemistry (Fig. 2K,O). It is essential to note that APP is physiologically expressed in the cytoplasm of cardiomyocytes. Lipofuscin granules were also stained with the anti-APP (except for antibodies targeting specifically Aβ epitopes; Fig. 3A–E) and anti-APLP2 antibodies (Fig. 3F,G). Actin, desmin, myosin and filamin C were not detected in BD-lesions (Suppl. Table 2). Accordingly, the cytoplasmic BD-inclusions in cardiomyocytes consisted of sAPPδ_373_, sAPPη or shorter N-terminal fragments of APP, which were PAS-positive (presumably glycosylated) and colocalized with ubiquitin, ubiquilin and p62/SQSTM1-positive material. TDP43, Aβ, abnormal phosphorylated τ-protein, desmin and other proteins listed in Suppl. Table 2 were not found in the aggregates. To confirm the presence of N-terminal APP-epitopes in BD-lesions we also stained the left ventricle myocardial sections of the rest of the 62 cases with anti-APP (22C11) which is directed against an N-terminal epitope of APP. In all cases with BD-lesions detected with anti-p62/SQSTM1 and the PAS-staining we also saw anti-APP-positive BD-lesions further confirming that BD-lesions represent aggregates of N-terminal APP-fragments. Cases free of BD-lesions in the PAS and anti-p62/SQSTM1-staining did not exhibit anti-APP-positive cardiomyocyte inclusions.

**Table 1:** Distribution of age, gender, clinical and pathological diagnoses.

		Data available in n cases
Age in years (mean/range)	57,5/0–85	62
Gender (M/F)	45/17	62
Atrial fibrillation (y/n)	9/42	51
Arterial hypertension (y/n)	26/36	62
Myocardial infarction (y/n)	9/53	62
Heart weight/g (mean/range)	410/8–850	62
Degree of cardiac fibrosis (mean/range)	1.56/0–3	62
Degree of hypertension-related cardiac fibrosis (mean/range)	0,694/0–3	62
p62-positive BD inclusions/mm² (left ventricle) (mean/range)	0.084/0–0.804	62
Diabetes mellitus (y/n)	9/53	62
Hyperlipoproteinemia (y/n)	10/28	38
Obesity (y/n)	15/23	38
Intracerebral hemorrhage (y/n)	8/54	62
Cerebral infarction (y/n)	16/46	62
Athersclerosis (mean/range)	31,25%/0–100%	59
Stage of SVD (mean/range)	1.6/0–3	62
Severity grade of CAA (mean/range)	0,23/0–2	62
Aβ-phase (MTL) (mean/range)	0.65/0–4	62
Braak NFT-stage (mean/range)	1.18/0-4	62
CERAD-score (mean/range)	0.13/0–2	62
NIA-AA degree of AD (mean/range)	0.39/0–2	62

Numbers of cases, age-spectrum, cardiac and AD-related pathologies and parameters, arterial hypertension, diabetes mellitus, atherosclerosis, cerebral small vessels disease (SVD), CAA, and heart weights: Mean values and ranges. The list of the individual cases is provided in Supplementary Table 1.

**Figure 4:**
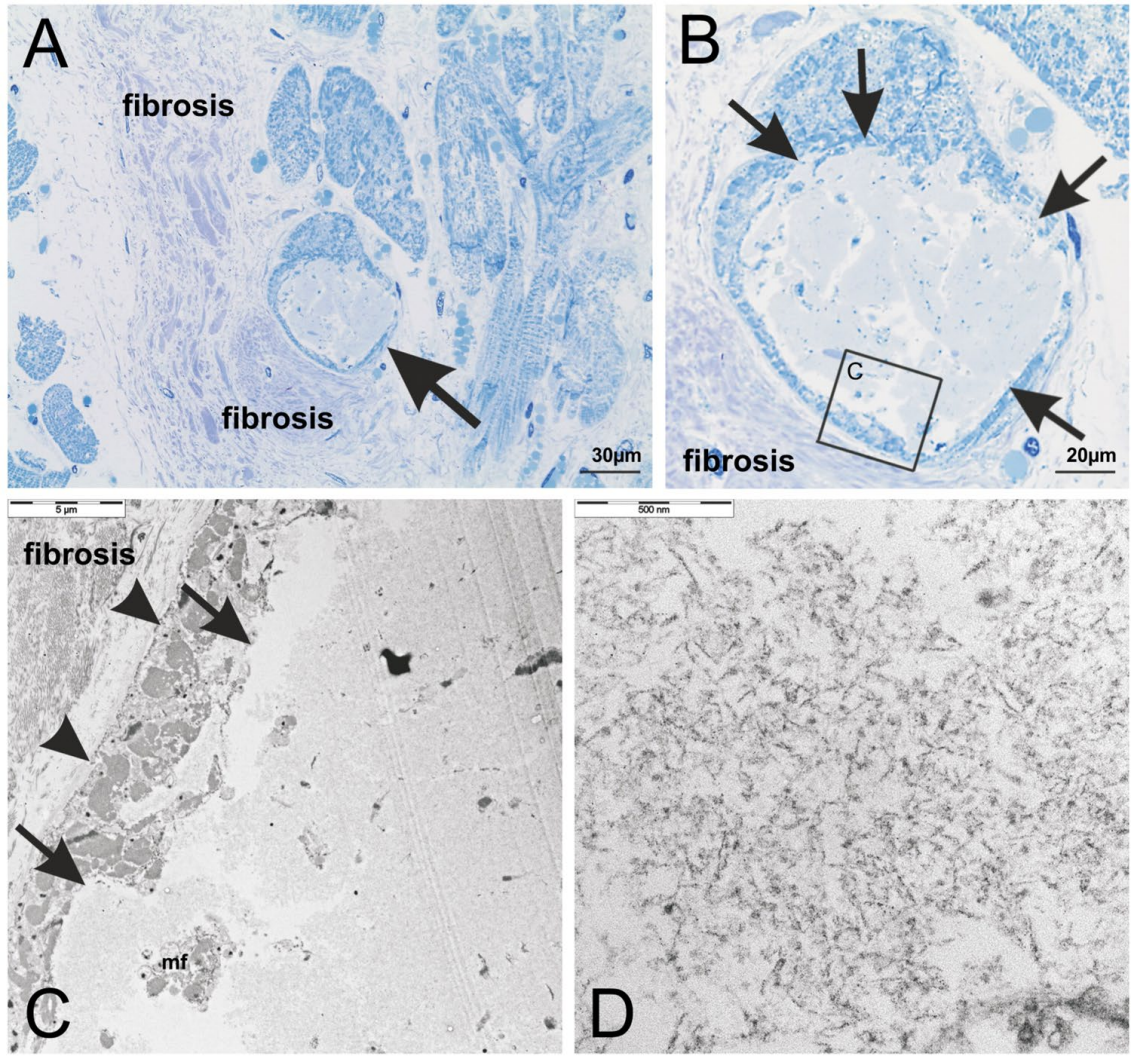
(**A**) Structural analysis of cytoplasmic BD-inclusions in a semithin section of the left ventricular myocardium of case No. 53 stained with methylene blue. The inclusion body (arrow) was located intracellular in the sarcoplasm of a transversally cut cardiomyocyte and its morphological appearance identified this inclusion as BD-inclusion as described previously^5^. Cardiomyocytes were surrounded by cardiac fibrosis consisting of collagen fiber tissue. (**B**) Enlarged section of (**A**) The inclusion body was sharply delineated (arrows) and contained aggregates of amorphous material. The boxed area indicates the part of the cardiomyocyte depicted in **C** at the ultrastructural level. (**C**,**D**) The BD-inclusion was also analyzed in ultrathin sections by electron microscopy. The aggregates were not membrane-coated and sharply demarcated from surrounding myofibrils (**C**: arrows). Between the aggregates only few myofibrils (**C**: mf) and no preserved cell organelles (**D**) could be seen. The aggregated material exhibited a fibrillar pattern at the higher magnification level (**D**). The cell membrane of the cardiomyocyte was intact (**C**: arrow heads) and surrounded by cardiac fibrosis. This ultrastructural pattern confirmed these lesions to represent BD.

To document that the BD-lesions identified here by histological criteria fit with the ultrastructural pattern of BD^5^ we performed electron microscopy in case No. 53 (Suppl. Table 1). The BD-lesions were identified according to its structural appearance in methylene blue-stained semithin sections as lesions consisting of amorphous material with a rim of myofibrils in the periphery (Fig. 4A,B)^5^. In ultrathin sections electron microscopy confirmed that BD-lesions contained fibrillar material (Fig. 4C,D). This material was not coated by a membrane and sharply bordered from adjacent myofibrils. Only few myofibrils and organelles were seen between the fibrillar aggregates (Fig. 4C). Lipofuscin-like material was not observed inside the BD-lesion (Fig. 4C,D).

### Distribution of BD-inclusions in the heart

**Figure 5:**
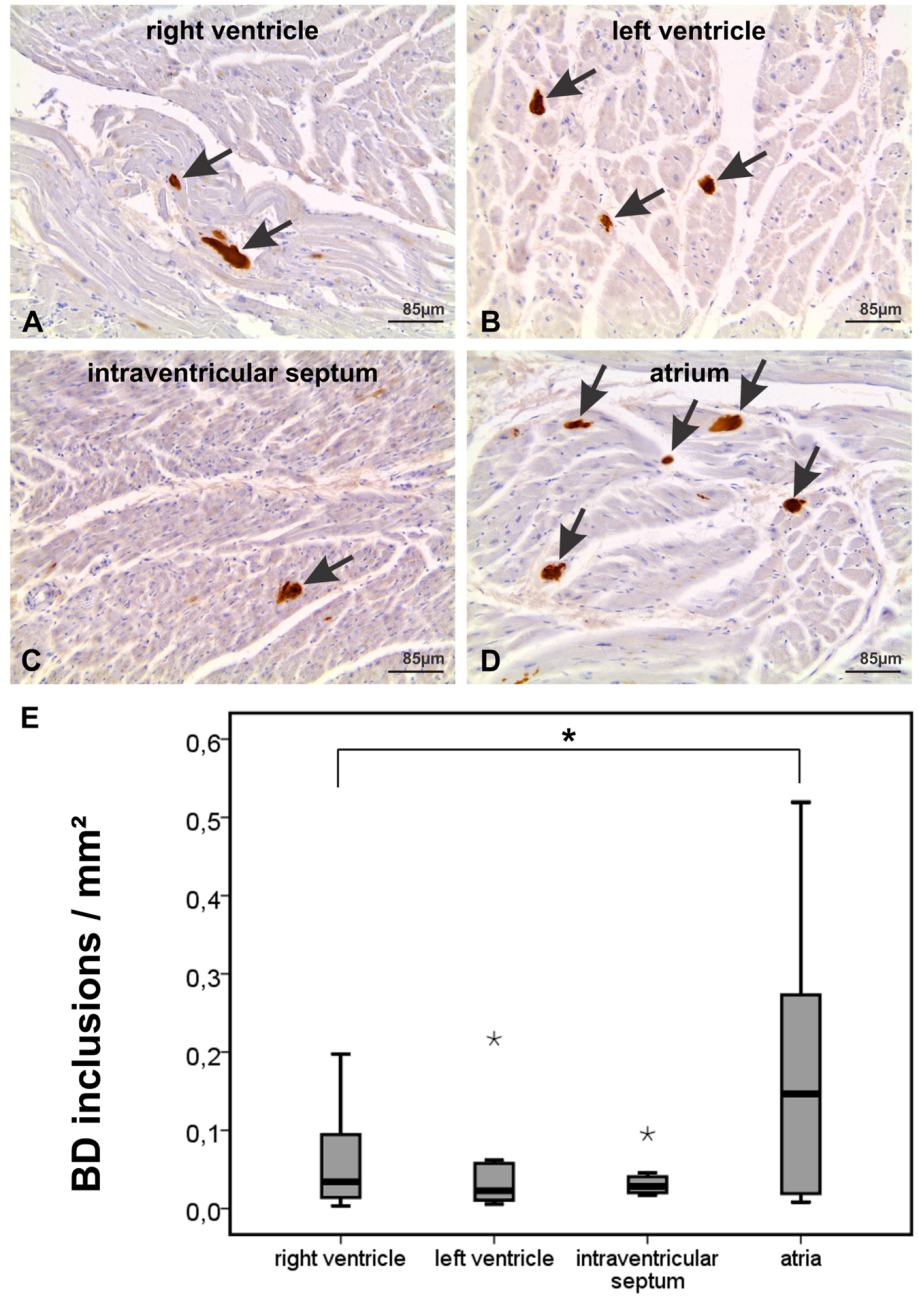
p62/SQSTM1-immunopositive BD inclusions were found in muscle cells of all parts of the heart of case No. 21 shown in transversal sections of the atrium, interventricular septum, and the left and right ventricles. (**A**) Two p62/SQSTM1-positive BD-inclusions are seen in this figure of the right ventricle (arrows). (**B**) Three BD-inclusions are marked with anti-p62/SQSTM1 in this image of the left ventricle (arrows). (**C**) The intraventricular septum image shows one BD-lesion (arrow). (**D**) The section of the atrium depicts five p62/SQSTM1-positive BD-lesions (arrows) indicating the high frequency of BD-lesions that could be detected in the atria by immunohistochemistry. The lesions were disseminated over the area of the atrial myocardium. (**E**) Assessment of the frequency of p62/SQSTM1-positive BD-inclusions in immunostained gross sections of the atria, ventricles and the intraventricular septum in cases No. 17, 21, 30, 33, 39, 44, 52, and 55 revealed the highest amount of inclusions in the atria and the lowest in the interventricular septum. The boxplot diagram shows the distribution of BD-inclusions/mm^2^ in the right and left ventricle, the interventricular septum and the atria (*statistical outliers, *significant at the 0.05 level (single sided Wilcoxon rank sum test), n = 8). The negative staining control showing only the staining with the secondary antibody while omitting the primary one is shown in Fig. 3 (**I**). The positive control for the p62/SQSTM1 antibody is depicted in Suppl. Fig. 1A.

To document the anatomical distribution of BD-lesions throughout the heart we embedded gross sections (6,5 × 4,8 cm of size) of the apex, left and right ventricle including the intraventricular septum, and the atria of 8 cases in paraffin, stained paraffin sections with hematoxylin and eosin, and immunostained them with anti-p62/SQSTM1 antibodies. After demonstration that the immunostaining for p62/SQSTM1 detects BD-lesions in the heart (Fig. 2A–C) and that it provides an easy detection of the BD-lesions (Fig. 5A–D) we used anti-p62/SQSTM1 immunohistochemistry for assessing the frequency of BD-lesions in a given part of the heart. The gross sections of the heart covered transverse sections of the entire heart a) at the level of the atria including both atria and the septum intraatriale and b) at the ventricular level covering both ventricles including the intraventricular septum and papillary muscles, and c) at the level of the apex of the heart. Using these sections, p62/SQSTM1-positive BD-inclusions in cardiomyocytes were observed in both ventricles and atria (Fig. 5A–D). The highest number of affected cardiomyocytes was seen in the atria and the lowest in the intraventricular septum (Fig. 5E). The number of atrial BD-inclusions was generally higher than that in ventricular areas (Wilcoxon rank sum test (single sided): p < 0.05). BD-inclusions were mainly found in the myocardial parenchyma outside the conduction system although the muscle fibers of the conduction system were not spared.

In the available samples of the quadriceps femoris muscle and/ or the diaphragm (n = 30), we wanted to clarify whether BD-lesions were limited to the heart or were also prevalent in the skeletal muscle as well. Using the PAS and anti-p62/SQSTM1-stainings we did not observe BD-lesions in the quadriceps femoris muscle or in the diaphragm of these individuals.

### Prevalence of cardiac BD-inclusions with age

**Figure 6:**
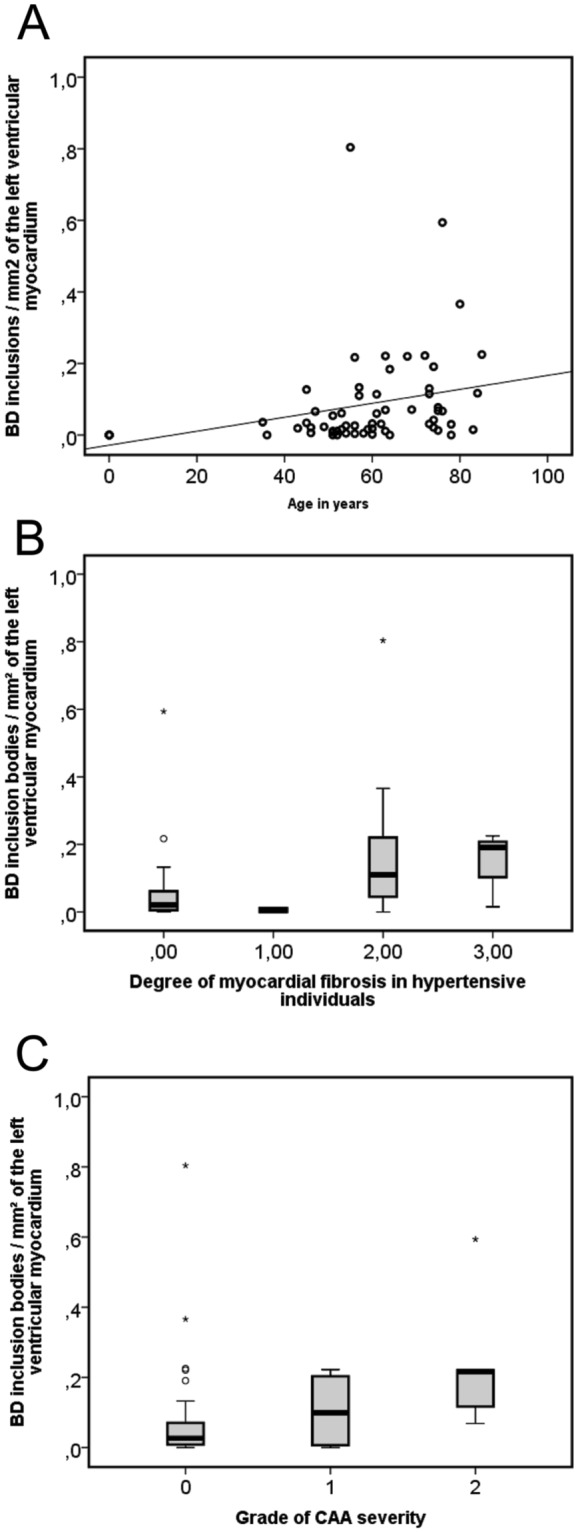
The frequency of p62/SQSTM1-immunopositive BD-inclusions/mm^2^ of the left ventricular myocardium was compared to age (**A**) the degree of myocardial fibrosis in hypertensive individuals (**B**) and CAA severity (**C**). (**A**) The frequency of inclusions increased very mildly in correlation with the age as demonstrated in a scatter diagram (Linear regression analysis: R² = 0.074; β = 0.273, p = 0.032, n = 62, Table 2) (black line = regression line). (**B**) An association of p62/SQSTM1-positive BD-inclusions of the left ventricle with the degree of myocardial fibrosis in hypertensive individuals is shown in this boxplot diagram (Linear regression analysis controlled for age and gender: R² = 0.163; β = 0.302, p = 0.032, n = 62, Table 2). (**C**) The boxplot diagram shows the association between the frequency of left ventricular BD-inclusions and the CAA severity (Linear regression analysis controlled for age and gender: R² = 0.16; β = 0.273, p = 0.035, n = 62, Table 2). (**B**,**C**) ° and *statistical outliers).

To study the frequency of BD-lesions in the heart we chose the left ventricle because previous studies reported the highest scores for BD-lesions in the left ventricle^6^ and because tissue blocks from left ventricle cross sections were available in all cases included in this study. p62/SQSTM1-positive BD-inclusions bodies were observed in left ventricle samples of individuals ranging from 35–85 years of age; patients younger than 35 (n = 4) did not exhibit p62/SQSTM1-positive BD inclusions. Five out of 54 cases between 35–78 years of age did not exhibit BD-inclusions in cardiomyocytes (Suppl. Table 1). The frequency of p62/SQSTM1-immunopositive BD-inclusions per mm² left ventricle section increased slightly with age (Linear regression analysis: R² = 0.074, β = 0.273, p = 0.023; Fig. 6A, Table 2).

**Table 2:** Statistical analysis: Binary logistic (*) and linear regression analysis with the frequency of p62/SQSTM1-BD inclusions as dependent variable.

n	Age in years	Gender (M/F)	Atrial fibrillation (y/n)	Arterial hypertension (y/n)	Myocardial infarction (y/n)	Heart weight/g	Degree of cardiac fibrosis	Degree of cardiac fibrosis in hypertensive individuals	Diabetes mellitus (y/n)		
	62	62	51	62	62	62	62	62	62		
p62/SQSTM1-positive BD inclusions: binary logistic (*) and linear regression (only one independent variable)	**β = 0.273;** **p = 0.032;** **R** ^**²**^ ** = 0.074**	p = 0.469*	p = 0.055*	p = 0.065*	p = 0.922*	p = 0.094	**β = 0.270;** **p = 0.033;** **R** ^**²**^ ** = 0.073**	**β = 0.332;** **p = 0.008;** **R** ^**²**^ ** = 0.11**	p = 0.652*		
p62/SQSTM1-positive BD inclusions: linear regression controlled for age and gender	n.d.	n.d.	n.d.	n.d.	n.d.	n.d.	p = 0.203	**β = 0.302;** **p = 0.032;** **R** ^**²**^ ** = 0.163**	n.d.		
p62/SQSTM1-positive BD inclusions: linear regression controlled for myocardial infarction and heart weight	n.d.	n.d.	n.d.	n.d.	n.d.	n.d.	n.d.	**β = 0.329;** **p = 0.009;** **R** ^**²**^ ** = 0.156**	n.d.		
p62/SQSTM1-positive BD inclusions: linear regression controlled for diabetes mellitus, obesity and atrial fibrillation	n.d.	n.d.	n.d.	n.d.	n.d.	n.d.	n.d.	**p = 0.17**	n.d.		
p62/SQSTM1-positive BD inclusions: linear regression controlled for CAA severity	n.d.	n.d.	n.d.	n.d.	n.d.	n.d.	n.d.	**β = 0.304;** **p = 0.004;** **R** ^**²**^ ** = 0.233**	n.d.		
p62/SQSTM1-positive BD inclusions: linear regression controlled for age, gender, and cardiac fibrosis in hypertensive individuals	n.d.	n.d.	n.d.	n.d.	n.d.	n.d.	n.d.	**β = 0.367;** **p = 0.007;** **R** ^**²**^ ** = 0.26**	n.d.		
p62/SQSTM1-positive BD inclusions: linear regression controlled for myocardial infarction, heart weight, and cardiac fibrosis in hypertensive individuals	n.d.	n.d.	n.d.	n.d.	n.d.	n.d.	n.d.	**β = 0.333;** **p = 0.005;** **R** ^**²**^ ** = 0.267**	n.d.		
**n**	**Hyperlipoproteinemia (y/n)**	**Obesity (y/n)**	**Intracerebral hemorrhage (y/n)**	**Cerebral infarction (y/n)**	**Athersclerosis in CaW (% of affected vessels)**	**Stage of SVD**	**Severity grade of CAA**	**Aβ-phase (MTL)**	**Braak NFT-stage**	**CERAD-score**	**NIA-AA degree of AD**
	**38**	**38**	**62**	**62**	**59**	**62**	**62**	**62**	**62**	**62**	**62**
p62/SQSTM1-positive BD inclusions: binary logistic (*) and linear regression (only one independent variable)	p = 0.488*	p = 0.404*	p = 0.247*	p = 0.181*	p = 0.161	p = 0.545	**β = 0.340;** **p = 0.007;** **R** ^**²**^ ** = 0.116**	p = 0.094	p = 0.166	p = 0.137	p = 0.072
p62/SQSTM1-positive BD inclusions: linear regression controlled for age and gender	n.d.	n.d.	n.d.	n.d.	n.d.	n.d.	**β = 0.273;** **p = 0.035;** **R** ^**²**^ ** = 0.16**	n.d.	n.d.	n.d.	n.d.
p62/SQSTM1-positive BD inclusions: linear regression controlled for myocardial infarction, degree of myocardial fibrosis and heart weight	n.d.	n.d.	n.d.	n.d.	n.d.	n.d.	**β = 0.337;** **p = 0.006;** **R** ^**²**^ ** = 0.166**	n.d.	n.d.	n.d.	n.d.
p62/SQSTM1-positive BD inclusions: linear regression controlled for diabetes mellitus, arterial hypertension and atrial fibrillation	n.d.	n.d.	n.d.	n.d.	n.d.	n.d.	**β = 0.306;** **p = 0.026;** **R** ^**²**^ ** = 0.137**	n.d.	n.d.	n.d.	n.d.
p62/SQSTM1-positive BD inclusions: linear regression controlled for myocardial fibrosis in hypertensive individuals	n.d.	n.d.	n.d.	n.d.	n.d.	n.d.	**β = 0.308;** **p = 0.003;** **R** ^**²**^ ** = 0.233**	n.d.	n.d.	n.d.	n.d.
p62/SQSTM1-positive BD inclusions: linear regression controlled for age, gender, and cardiac fibrosis in hypertensive individuals	n.d.	n.d.	n.d.	n.d.	n.d.	n.d.	**β = 0.334;** **p = 0.008;** **R** ^**²**^ ** = 0.26**	n.d.	n.d.	n.d.	n.d.
p62/SQSTM1-positive BD inclusions: linear regression controlled for myocardial infarction, heart weight, and cardiac fibrosis in hypertensive individuals	n.d.	n.d.	n.d.	n.d.	n.d.	n.d.	**β = 0.339;** **p = 0.005;** **R** ^**²**^ ** = 0.267**	n.d.	n.d.	n.d.	n.d.

The independent variables are the variables in the respective columns and the control variables are provided for each line. n provides the number of cases available for the variables in the columns used to calculate linear regression analysis.

Statistical analysis of the effects on BD by age, gender, cardiac and AD-related pathologies and parameters, arterial hypertension, diabetes mellitus, atherosclerosis in the circle of Willis (CaW), cerebral small vessels disease (SVD), CAA by binary logistic (*) or linear regression analysis. R² reflects the degree in how far a respective model term of independent variables explains the dependent variable. β represents the standardized β-coefficient for each individual independent variable. Abbreviations: *m/f* Male/Female.

### Association of cardiac BD-inclusions with cardiac fibrosis in hypertensive individuals, and cerebral amyloid angiopathy (CAA)

To clarify whether BD is related to a cardiovascular disease or risk factor we studied its association with arterial hypertension, myocardial infarction, atrial fibrillation, cardiac fibrosis, heart weight, diabetes mellitus, hyperlipoproteinemia, obesity, cerebral hemorrhage, cerebral infarction, cerebral small vessel disease, atherosclerosis of the circle of Willis, and CAA by using linear regression analysis with model terms including one or more independent variables. The number of p62/SQSTM1-positive BD-inclusions/mm² in the left ventricle was associated with the degree of myocardial fibrosis (Linear regression analysis – myocardial fibrosis: R² = 0.073, β = 0.270, p = 0.033, Table 2). This association was not seen after controlling the model term for age and gender (Linear regression analysis: p = 0.203, Table 2). When focusing on the degree of myocardial fibrosis in individuals with arterial hypertension and including non-hypertensive cases as controls BD was associated with the degree of myocardial fibrosis in hypertensive individuals (Fig. 6B; Linear regression analysis: R² = 0.11, β = 0.332, p = 0.008, Table 2). After controlling the model term for a) age and gender (Linear regression analysis: β = 0.302, p = 0.032; R² = 0.163, Table 2), or b) myocardial infarction, and heart weight (Linear regression analysis: β = 0.329, p = 0.009; R² = 0.156, Table 2) the R² values of the model increased. Morphologically, p62/SQSTM1-positive BD-inclusions were found close to areas of fibrosis.

The severity degree of CAA^24^ was associated with the frequency of left ventricular p62/SQSTM1-positive BD-inclusions (Fig. 6C; Linear regression analysis: R² = 0.116, β = 0.340, p = 0.007, Table 2). This association was also seen in a model term controlled for myocardial infarction, degree of myocardial fibrosis, and heart weight (Linear regression analysis: R² = 0.166, β = 0.337, p = 0.006, Table 2) as well as in a model controlled for age and gender (Linear regression analysis: R² = 0.16, β = 0.273, p = 0.035, Table 2). Interestingly, the effects of CAA severity and the degree of myocardial fibrosis in hypertensive individuals showed an independent influence on the frequency of p62/SQSTM1-positive BD-inclusions increasing the R² of the model term to R² = 0.267 when tested in one model term with CAA severity, degree of myocardial fibrosis in hypertensive individuals as independent variables controlled for myocardial infarction and heart weight in one model term (Linear regression analysis: R² = 0.267; CAA severity β = 0.339, p = 0.005; degree of myocardial fibrosis in hypertensive individuals β = 0.333, p = 0.005, Table 2) as well as when controlled for age and gender in the second (Linear regression analysis: R² = 0.26; CAA severity β = 0.334, p = 0.008; degree of myocardial fibrosis in hypertensive individuals β = 0.367, p = 0.007, Table 2).

No associations of the frequencies of BD-inclusions of the left ventricle were seen with (a) the stage of cerebral small vessel disease distribution (p = 0.545)^25^, (b) the percentage of vessels of the circle of Willis being macroscopically affected by atherosclerosis (p = 0.161)^26^, (c) the presence of myocardial infarction (p = 0.922), (d) presence of atrial fibrillation (p = 0.055), (e) arterial hypertension (p = 0.065), (f) diabetes mellitus (p = 0.652), (g) obesity (p = 0.404), (h) hyperlipoproteinemia (p = 0.488), and (i) heart weight (p = 0.094) as observed by binary logistic and linear regression models (Table 2).

### No association of cardiac BD-inclusions with AD pathology

To clarify whether p62/SQSTM1-positive BD-inclusions in cardiomyocytes exhibiting N-terminal epitopes of APP are related to AD-pathology linear regression analysis was calculated for the frequency of cardiomyocytes with p62/SQSTM1-positive BD-inclusions of the left ventricle as independent variable and the phases of Aβ plaque pathology in the medial temporal lobe (MTL)^27^, the Braak-neurofibrillary tangle (NFT) stages^28^, the score of neuritic plaque pathology as recommended by the Consortium to establish a registry for AD (=CERAD-score)^29^, and the degree of AD-pathology according to the National Institute of Aging-Alzheimer’s Association recommendations^30^ as respective dependent variables. No association was found (p ≥ 0.072) (Table 2).

## Discussion

Here, we show that inclusions in cardiomyocytes known as BD with the typical histopathological and ultrastructural pattern are detectable with antibodies directed against the N-terminus of APP. This accumulation of APP cleavage products is associated with the severity of CAA in leptomeningeal and cortical blood vessels. CAA is histopathologically characterized by the deposition of Aβ, which is another cleavage product of APP (Fig. 1A) and represents a cerebral vessel disorder known as the 2^nd^ most frequent cause of hemorrhage in the elderly brain after hypertension-related bleedings^31–33^. This association as well as the involvement of APP in both pathologies may point to a possible molecular link between both pathologies. However, no significant association was seen with AD-related amyloid plaque pathology as represented by the amyloid plaque phases in the MTL^27^ as well as with neuritic plaque pathology as assessed by the CERAD-score for neuritic plaque pathology^29^ and with NFT-pathology assessed by the Braak NFT-stages^28^.

In the BD-lesions, the aggregated N-terminal APP fragments colocalize ubiquitin, p62/SQSTM1, and PAS-positivity, presumably indicative for the presence of glycosylated proteins such as APP that could be ubiquitinated and linked to p62/SQSTM1, as demonstrated by triple label immunofluorescence and by assessing subsequent sections. To classify these APP fragments, we used antibodies detecting the Aβ-region of APP as well as epitopes N-terminal to the β-secretase cleavage site of APP but C-terminal to the δ_373_- and η-secretase cleavage site (D- and M-epitopes of APP)^18^ (Fig. 1). Only the 22C11 and the 9023 APP antibodies – both detecting sAPPδ/η – stained cytoplasmic inclusion bodies whereas all other antibodies, including those against APP-epitopes C-terminal to its δ and η-cleavage sites, remained negative (Fig. 1). Since the 22C11 antibody crossreacts with APLP2 we excluded APLP2 as possible component in the BD-lesions by double label immunofluorescence detecting p62/SQSTM1 and APLP2. Accordingly, the material that accumulates in BD-lesions and colocalized PAS-positivity, p62/SQSTM1, and ubiquitin appears to represent aggregates of sAPPη, sAPPδ_373_, or another N-terminal APP fragment with its C-terminus N-terminal to the η-secretase cleavage site (Fig. 1).

BD cytoplasmic inclusion bodies of heart muscle cells in the majority of elderly individuals are different from other cardiomyopathies including those with inclusions containing mutant proteins such as desmin or valosin-containing protein. Nuclear inclusions and similar aggregates in the skeletal muscle were not observed as it is expected in IBMPFD^2^ or desmin-related myopathies^1^. The fact that BD lesions were not associated with the clinical diagnosis “glycogenosis” and that they contained aggregates of sAPPδ/η argue against a disorder of glycogen metabolism as considered by some authors^5,7^. An association with diabetes mellitus, obesity and hyperlipoproteinemia was not found in our cases. Moreover, the fact that presenilin 1/2 (i.e. the active center of the γ-secretase^34^) mutations can cause cardiomyopathies^35^ and that Aβ can cause degeneration of cardiomyocytes^22^ may further argue in favor of a critical function of APP and/or its cleavage products in the heart. The presence of APP in the BD-lesions also argues against impaired autophagy as primary cause of BD because other protein aggregation disorders that accumulate p62/SQSTM1-positive material can be driven by specific mutations of the accumulating protein such as mutations in the α-synuclein gene in Parkinson’s disease^36^ or in the TDP43 gene (*TARDBP*) in amyotrophic lateral sclerosis (ALS)^37^. As such, it is tempting to speculate that APP and the accumulation of its N-terminal fragments is critically involved in BD-related cardiomyocyte degeneration, probably similar to the role of α-synuclein in sporadic Parkinson’s disease or TDP43 in sporadic ALS.

p62/SQSTM1-positive sAPPδ/η aggregates in cardiomyocytes representing BD are associated with age confirming previous studies on BD^3,5–7^ and with myocardial fibrosis in the left ventricle of hypertensive individuals. A significant association with heart weight, myocardial infarction, arterial hypertension alone or atrial fibrillation could not be seen. Previous studies on BD already discussed a frequent occurrence of these lesions in cases with cardiovascular diseases^3,6^. Some animal models with autophagic-lysosomal inhibition leading to increased p62/SQSTM1-levels show hypertension^38^ or cardiomyopathy^39,40^ further arguing in favor of a potential link between the accumulation of p62/SQSTM1-positive protein aggregates in cardiomyocytes with BD and hypertension or cardiomyocyte degeneration.

Recently, it was shown that spontaneously hypertensive stroke-prone rats (SHRSP) develop CAA^15,16^. This finding supports the association between a cardiac pathology which is related to arterial hypertension and CAA. Another, argument for a link between hypertension-related cardiac pathology and CAA is the finding of Gerth *et al*. that a distinct group of CAA cases based upon the parenchymal/vascular distribution pattern of modified Aβ forms was associated with arterial hypertension^41^. These results from other studies further support the hypothesis that hypertension-related lesions of the heart and CAA are linked.

The distribution of BD-lesions in all parts of the heart without specific affection of the conduction system confirms earlier studies^3,6^ and indicates that this pathology may affect the function of the entire heart. Earlier studies based upon H&E-stained tissue samples showed slight differences in the distribution over the heart. In these H&E-based studies, the most BD-lesions were found in the septum and the ventricles whereas they appeared less abundant in the atria^3,6^. Since we used immunohistochemistry for the specific detection of the lesions our findings may be less biased than the studies from 1935^3^ and 1955^6^ because of superior methodology for the detection of the BD-lesions, which is now available with highly specific immunohistochemical markers such as anti-p62/SQSTM1 and anti-APP antibodies. This enables us now to define BD-lesions as p62/SQSTM1-positive aggregates of sAPPδ/η and allows more precise correlation analyses with disorders potentially related with these lesions.

In summary, we describe BD-lesions to represent inclusions of sAPPδ/η aggregates in cardiomyocytes. BD appears, thereby, to represent an age-related protein aggregation pathology in the heart associated with CAA and myocardial fibrosis in individuals with arterial hypertension. Since APP is involved in both CAA and BD a molecular link appears possible as well as a link with hypertension-associated cardiomyocyte alterations.

It will be also important to be aware of BD in the pathological differential diagnosis of inclusion body cardiomyopathies with p62/SQSTM1- or ubiquitin-positive inclusions.

## Methods

All methods have been performed in accordance with the relevant guidelines and regulations.

### Pathology

Heart and brain tissue of 62 autopsy cases was studied (Table 1, Suppl. Table 1). Quadriceps femoris muscle and/or diaphragm were available for 30 of these cases. The heart weight was obtained at autopsy.

The age-range of the sample was 0–85 years. Cases with and without heart and/or brain disorders were included (Table 1, Suppl. Table 1). The autopsy tissue has been collected by the Ulm University Brain Bank at the Institute of Pathology with approval of the local Ethics Committee (Decision-No. 378/13). The autopsies were performed with informed consent of the next in kin. Cause of death included cancer (n = 4), cardiovascular disease (n = 28), respiratory failure (n = 18), neurological reasons (n = 8), multiple organ dysfunction (n = 1), and infectious diseases (n = 3). Inclusion criteria were the availability of autopsy tissue from brain and heart of a given case. Exclusion criteria were spongiform encephalopathies, tuberculosis, HIV, hepatitis B and C infections. The reason for excluding cases with these infectious disorders was the safety in the research laboratory.

Tissue samples were formalin fixed and embedded in paraffin. Left ventricle samples covering cross-sections through the myocardium of all hearts were stained. Brain pathology was studied as previously published^42^. 5 µm thick sections from all cases were cut and stained with the hematoxylin and eosin, Masson-Goldner, and periodic-acid Schiff (PAS) staining techniques. Immunohistochemistry was performed with antibodies listed in Supplementary Table 2. All cases were thereby stained with antibodies against p62/SQSTM1 and the N-terminus of APP (22C11) whereas the other antibodies were only used in cases No. 1, 15, 17, 50, 53, 56 and 58 to illustrate the expression pattern of the BD-lesions with the antibodies listed in Suppl. Table 2. For visualization primary antibodies were either detected with Carbocyanin 2, Carbocyanin 3, or Carbocyanin 5-marked secondary antibodies or with biotin-labeled secondary antibodies, avidin-biotin complex and diaminobencidine-HCl or the DAKO-Real alkaline phosphatase/RED substrate kit (DAKO, Glostrup, Denmark). In the event that we combined two mouse antibodies in a triple staining, the second primary mouse antibody was linked to biotinylated Fab-fragments against mouse IgG prior to the incubation with the sample and Carbocyanin 2-conjugated streptavidin was used for visualization^43^. Positive and negative controls were carried out. Light and fluorescence microscopy was performed with a Leica DMLB microscope (Leica, Germany). Pictures were taken with Leica DFC 290 and DFC 7000 T digital cameras (Leica, Germany) and were processed for contrast and colocalization overlays with CorelPhotopaint XVI. In images of triple labelled sections, the red-orange Carbocyanin 3 signal was pseudo-coded in blue.

Heart pathology (infarcts, fibrosis, and conventional signs of cardiomyopathy), skeletal muscle pathology, and brain pathology (AD-related lesions) were assessed. The degree of cardiac fibrosis of the left ventricular myocardium was graded in 4 levels (0–3) based on the examination of Masson-Goldner stained sections: 0 = no fibrosis detectable, 1 = small solitary areas of fibrosis (no more than 3 areas of fibrosis in the size of no more than 3 muscle fiber diameters), 2 = diffuse or multiple (>3 per section) areas of fibrosis, and 3 = general diffuse fibrosis (an area of more than 20% of the section was covered by fibrosis).

For electron microscopy formalin-fixed tissue of case No. 53 was embedded in Epon after osmium and uranyl acetate staining. Semi- and ultrathin sections were cut. Semithin sections were stained with methylene blue. For electron microscopy a JEM-1400 electron microscope (JEOL, Tokyo, JP) was used and grids were block stained with lead citrate.

To determine the distribution of the p62/SQSTM1-positive BD-inclusions in cardiomyocytes we embedded gross sections of the apex, left and right ventricle including the intraventricular septum, and both atria with septum intraatriale of 8 cases (cases No. 17, 21, 30, 33, 39, 44, 52, and 55) in paraffin, stained paraffin sections with H&E, and immunostained them with anti-p62/SQSTM1 antibodies. For semiquantitative assessment of the density of BD-inclusions we counted the number of p62/SQSTM1-positive inclusions in the entire myocardium covered in a given section from the left ventricle (all cases), the right ventricle, the atria and the intraventricular septum (from the 8 cases processed for paraffin macrosections of the latter 4 regions) and measured the area of the respective myocardial tissue with the ImageJ image analysis software (NIH, Bethesda, USA). A total area of 1–3 cm² myocardium was assessed per case and region depending on the size of the embedded samples. These data were used to calculate the BD-inclusion frequency/mm^2^.

Neuropathologically, the degree of AD pathology^30^, the phase of Aβ deposition in the MTL^27^, the stage of NFT-pathology^28^, the CERAD-score of neuritic plaque pathology^29^, the expansion of atherosclerosis in the Circle of Willis^26^, and the stages of cerebral small vessel disease^25^ were assessed as described previously. The grade of cerebral amyloid angiopathy (CAA)-related vessel wall destruction, i.e. the CAA severity, has been rated according to Vonsattel *et al*.^24^: 0 = no CAA; 1 = Aβ-positive material in vessel wall(s) without significant smooth muscle cell degeneration; 2 = Aβ-positive material in vessel wall(s) with significant destruction and fibrosis of media; and 3 = Aβ-positive material in vessel wall(s) with micro- or macrohemorrhages.

### Assessment of clinical characteristics

Clinical data were analyzed retrospectively (Suppl. Table 1). The clinical protocols and the autopsy reports provided information about a history of arterial hypertension, diabetes mellitus, hyperlipoproteinemia, obesity, coronary heart disease, myopathies, clinically evident episodes of embolic diseases, atrial fibrillation, anticoagulation treatment, brain disorders as well as other diseases and the pathologically-confirmed cause of death. Myocardial and cerebral infarction, brain pathology including AD-lesions and other findings were taken from the autopsy reports.

Electrocardiograms, heart ultrasound reports, heart magnetic resonance imaging, myocardial positron emission tomography and coronary angiography reports were screened in the event that these diagnostic tools were applied prior to death and were, in so doing, available only in a subset of the cases. The clinical diagnosis of a distinct heart disease was noted. Cases without clinical signs of heart disease during lifetime were considered to have no clinically apparent signs of heart disease (Suppl. Table 1).

### Statistical analysis

Statistical analysis was carried out by using binary logistic as well as linear regression analysis. For binary logistic and linear regression analysis we used in the first step a model term with one dependent and one independent variable. In the event that this analysis revealed a significant association we included further independent variables into the model term to control for, e.g. age and gender. To avoid collinearity effects we limited the maximum number of independent variables to four. The R²-values provided by linear regression analysis refer to the entire model term (including all independent variables) and give information up to which extend a given model term describes the dependent variable. The effects of the independent variables were provided by the respective standardized β-coefficients and p-values.

For comparing frequencies of BD-lesions among different parts of the heart (atria, left and right ventricles and intraventricular septum) the Wilcoxon rank sum test was used. Calculations of the statistical tests were performed with IBM-SPSS 24 software.

### Ethics

Ethical approval by the Ulm University Ethics Committee (Decision-No. 378/13). An informed consent for autopsy and scientific use of autopsy tissue with clinical information was granted for all cases included. All methods have been performed in accordance with the relevant guidelines and regulations.

## Electronic supplementary material

Supplementary Material

## Data Availability

All cases are listed in supplementary table with measurements obtained throughout this study, with the main clinical diagnosis, age and gender. Due to legislation and privacy protection any medical reports and files of the cases included in this study cannot be made available.
