# Supplementary material for: Amyloid precursor protein-fragments-containing inclusions in cardiomyocytes with basophilic degeneration and its association with cerebral amyloid angiopathy and myocardial fibrosis
Source: Sci Rep. 2018 Nov 9;8:16594. doi: 10.1038/s41598-018-34808-7 (PMC6226444; doi:10.1038/s41598-018-34808-7)

Supplementary material (Data, Tables, Figure)

Amyloid precursor protein-fragments-containing inclusions in cardiomyocytes with basophilic degeneration and its association with cerebral amyloid angiopathy and myocardial fibrosis

Lara Maria Krämer[1], Johannes Brettschneider[2], Jochen K. Lennerz[1,3], Daniel Walcher[4], Lubin Fang[5], Angela Rosenbohm[5], Karthikeyan Balakrishnan[1,6], Julian Benckendorff[1], Peter Möller[1], Steffen Just[4], Michael Willem[7], Albert C. Ludolph[5], Dietmar Rudolf Thal[1,8,9]

1 Institute of Pathology, Ulm University, Ulm, Germany
2 RehaClinic Lucerne, Switzerland
3 Department of Pathology, Massachusetts General Hospital/Harvard Medical School, Boston, MA, USA
4 Department of Internal Medicine II (Cardiology), Ulm University, Ulm, Germany
5 Department of Neurology, Ulm University, Ulm, Germany
6 Department of Gene Therapy, Ulm University, Ulm, Germany
7 Biomedical Center, Ludwig-Maximilians-Universität Munich, Germany
8 Department of Neuroscience and Leuven Brain Institute, KU-Leuven, Leuven, Belgium
9 Department of Pathology, UZ-Leuven, Leuven, Belgium

Legends:
Supplementary Table 1: List of cases. (Data)
Supplementary Table 2: List of proteins observed for its presence in p62/SQSTM1-positive BD inclusions in cardiomyocytes and the respective antibodies used for this purpose.
Supplementary Fig. 1: Positive controls for immunohistochemistry (**A-D** visualized with avidin-biotin complex and DAB, **E-H** visualized with carbocyanine (Cy) labelled secondary antibodies): **A**: p62/SQSTM1-staining of cytoplasmic dipeptide repeat inclusions in CA4 neurons (arrows) of a case with ALS carrying a C9Orf72 hexanucleotide mutation. The lacking nuclear signal serves as intrinsic negative control. **B, C**: Neuritic plaques (arrows) and less strongly the neuropil and the cytoplasm of neurons of AD brain (**B**: temporal neocortex; **C**: hippocampal sector CA1) are marked with the antibodies against the N-terminus of APP (22C11) (**B**) and the Aη region of APP (D-epitope antibody) (**C**). The nuclei are not stained and serve as intrinsic negative controls. **D**: The anti-Aβ_{1-17} antibody 6E10 clearly stains amyloid plaques (arrows) in AD temporal neocortex whereas the neighboring neuropil remains negative. **E, F**: Dipeptide inclusions in the granule cell layer of the cerebellum of an ALS case carrying the C9Orf72 hexanucleotide repeat mutation stained with the p62/SQSTM1 antibody visualized with Carbocyanin-5 (Cy5)-labelled secondary antibodies (**E**) and with the ubiquitin antibody-coupled on biotinylated anti-mouse Fab fragments and labelled with Cy2-conjugated streptavidin (**F**). The inclusions stained with both antibodies in this double labelled section are indicated by arrowheads. Ubiquitin antibodies also mark other lesions that are not detected by the p62/SQSTM1 antibody. **G**: The antibody against the M-epitope of the APP-Aη region stains a neuritic plaque with dystrophic neurites (arrowheads) in the parietal cortex of an AD brain (the original Cy 3 color is shown here; the image is not recoded into blue as it is done for triple label immunofluorescence figures!). **H**: Amyloid plaque (arrowhead) in the parietal cortex of an AD brain detected with the polyclonal Aβ antibody 3552 visualized with Cy3-labelled secondary antibodies (the original color is shown here; the image is not recoded into blue as it is done for triple label immunofluorescence figures!).

Supplementary Tab. 1: List of cases. (Data)

Case number	Age (years)	SEX	AβMTL phase	Braak NFT stage	CERAD score	NIA-AA degree of AD pathology	clinical diagnosis	pathological diagnosis	neuropathological diagnosis	CAA severity	Expansion of atherosclerosis in the cricle of Willis	SVD Stage	intracerebral hemorrhage	brain infarction	arterial hypertension	diabetes mellitus	Obesity	atrial fibrillation	myocardial infarction	degree of myocardial fibrosis	degree of hypertension-related myocardial fibrosis	BD (p62-positive) inclusion bodies / mm²	Heart weight	BMI	hyperlipoproteinemia	CDR	GDS	PMI
1	68	m	2	2	0	1	ALS, prostate carcinoma, artertial hypertension	ALS	ALS, SVD, hypoxemia in CA1	2	36,36	2	no	no	yes	no	yes	no	no	2	2	0,2201	365	n.k.	no	0	1	144
2	54	m	0	0	0	0	CLL, stem cell transplantation with GVHD, GBS, arterial hypertension, muscular atrophy	CLL, myocardial hypertrophy, pneumonia	Bergmann cell gliosis, neurogenic muscular atrophy, meningeoma anterior cervical root WHO°1	0	0,00	1	no	no	yes	no	no	no	no	2	2	0,0262	545	22,7	no	0	1	72
3	60	m	0	0	0	0	HD, kachexia, pneumonia	HD, pneumonia	HD	0	0,00	2	no	no	no	no	no	n.k.	no	0	0	0,0017	340	n.k.	n.k.	n.k.	n.k.	24
4	73	f	0	3	0	0	Carcinoma with unknown primary tumor, kachexia, osteolysis	plasmocytoma	extradural plasmocytoma, neurogenic muscular atrophy	0	45,45	1	no	no	no	no	no	n.k.	no	2	0	0,0312	280	n.k.	n.k.	n.k.	n.k.	24
5	76	f	0	2	0	0	Asthma bronchiale, pulmonary emphysema of the lung, ALS, arterial hypertension	pulmonary emphysema, atherosklerosis, ALS	ALS, AGD, neurogic muscular atrophy	0	50,00	3	no	no	yes	no	n.k.	no	no	2	2	0,0670	330	n.k.	n.k.	0	1	48
6	64	f	0	3	0	0	Spinocerebellar syndrome, sepsis-associated encephalopathy, arterial hypertention, polyarthrosis, CML?	CML, sepsis-induced multiple organ failure	PD	1	n.d.	2	no	no	yes	no	no	no	no	2	2	0,1841	280	24,0	no	0,5	1	12
7	51	m	0	0	0	0	Malignant melanoma, esophageal carcinoma, limbic encephalitis, pneumonia	Status after melanoma surgery, esophageal carcinoma, pneumonia	paraneoplastic limbic encephalitis	0	33,33	0	no	no	no	no	no	no	no	2	0	0,0083	400	n.k.	no	3	7	72
8	75	m	1	2	1	1	ALS	ALS	ALS, CAA, SVD	2	45,45	2	no	no	no	no	n.k.	no	no	0	0	0,0683	385	n.k.	n.k.	0	1	24
9	62	m	0	1	0	0	alcohol abuse, liver cirrhosis, carcinoma with unknown primary tumor	carcinoma	normal brain	0	9,09	2	no	no	no	no	no	no	no	2	0	0,0306	410	24,8	no	0	1	12
10	83	m	0	2	0	0	coronary heart disease, myocardial infarction, chronic renal insufficiency, atrial fibrillation	hypertensive heart disease, biventricular cardiac insufficiency, atherosclerosis, coronary heart disease	SVD, atherosclerosis, multiple old brain infarcts	0	81,82	3	no	yes	yes	no	no	yes	yes	3	3	0,0153	520	26,3	no	2	7	48
11	57	m	0	2	0	0	arterial hypertension, ALS	ALS	ALS	0	0,00	2	no	no	yes	no	yes	no	no	2	2	0,1056	525	30,1	no	0	1	96
12	56	m	0	0	0	0	CML	CML	normal brain	0	36,36	2	no	no	no	no	no	no	no	0	0	0,0257	350	25,8	no	0	1	48
13	58	m	0	1	0	0	GBS, status after varicosis surgery, pneumonia	pulmonary embolism	polyradiculitis, toxic myopathy	0	18,18	2	no	no	yes	no	yes	no	no	0	0	0,0049	530	30,7	no	n.k.	n.k.	48
14	47	f	0	1	0	0	multiple sclerosis, epileptic seizures, hemiplegia, intracerebral bleeding, obesity, hyperuricemia, nikotin abuse	pulmonary embolism, glomerulosclerosis and renal atherosclerosis	SDH, red brain infarct, atherosclerosis, multiple sclerosis lesions	0	33,33	2	yes	yes	no	yes	yes	no	no	0	0	0,0656	425	n.k.	no	n.k.	n.k.	72
15	61	m	1	2	0	1	ALS, pneumonia	ALS, respiratory failure	ALS	0	0,00	2	no	no	no	no	no	no	no	2	0	0,1141	340	n.k.	no	0	1	24
16	53	m	1	1	0	1	ARDS, pneumonia	pneumonia	SVD	0	0,00	2	no	no	no	no	no	no	no	2	0	0,0612	400	22,0	no	0	1	48
17	45	m	1	1	0	1	IgA-nephritis and terminal renal insufficiency, pneumonia, atherosclerosis	IgA-nephritis, status after renal transplantation, renal pulmonal embolism, pneumonia	mild SVD, inactivity related muscular atrophy	0	27,27	3	no	no	no	no	n.k.	no	no	2	0	0,1267	260	n.k.	n.k.	0	1	24
18	0,0301	m	0	0	0	0	hydrops fetalis, pulmonal hypoplasia	hydrops fetalis, vena cava thrombosis	amyoplasia congenita, microinfarcts in the basal ganglia, hippocampal signs of hypoxemia	0	0,00	0	no	yes	no	no	n.k.	no	no	2	0	0,0000	16,9	n.k.	n.k.	n.k.	n.k.	24
19	78	m	4	4	1	2	prostate carcinoma, subcortical vasculare encephalopathy with signs of vascular dementia, meningeoma, atrial fibrillation	atherosklerosis, coronary heart disease	AD, VD, meningeoma WHO°2	1	100,00	3	no	yes	yes	yes	yes	yes	no	1	1	0,0000	510	28,7	yes	3	7	48
20	78	f	1	1	0	1	subcortical vascular encephalopathy, dementia	circulatory insufficiency, old myocardila infarction, pulmonary emphysema	SVE, corticobasal degeneration, small bleeding near corpus callosum	0	45,45	3	yes	yes	yes	no	no	yes	yes	2	2	0,0305	290	20,8	yes	3	6	48
21	56	m	2	2	0	1	celiac disease, pneumonia, cerebellar degeneration and encephalopathy	celiac disease, spleen atrophy, pneumonia	Limbic- and brainstem encephalitis, old red cortical infarct	2	27,27	2	no	yes	no	no	n.k.	no	no	1	0	0,2170	275	n.k.	n.k.	1	6	24
22	43	m	0	1	0	0	Crohn's disease, acute myocardial infarction	sudden cardiac death	normal brain	0	9,09	0	no	no	no	no	n.k.	no	yes	2	0	0,0185	430	n.k.	n.k.	n.k.	n.k.	120
23	51	m	0	0	0	0	bladder carcinoma, signs of infection and cognitive deficits	bladder carcinoma, acute pancreatitis, coronary heart disease, pulmonary embolism	brain edema, mild SVD, neurogenic muscular atrophy with accompanied myopathic changes	0	36,36	2	no	no	no	no	n.k.	n.k.	no	2	0	0,0108	390	n.k.	n.k.	n.k.	n.k.	48
24	61	f	0	1	0	0	ALS, atrial fibrillation, arterial hypertension, central respiratory failure	ALS, respiratory failure	ALS	0	0,00	1	no	no	yes	no	yes	yes	no	2	2	0,0597	260	n.k.	yes	n.k.	n.k.	48
25	60	f	4	1	0	1	polyneuropathy, arterial hypertension, hyperlipidemia, hypothyreosis	coronary heart disease, pulmonary emphysema	lymphocytic encephalo-myelitis, inactivity-related muscular atrophy	0	54,55	2	no	no	yes	no	n.k.	n.k.	no	2	2	0,0180	290	n.k.	yes	n.k.	n.k.	96
26	56	m	0	1	0	0	pulmonary embolism, SDH and SAH, coronary heart disease, alcohol abuse, hepathopathy, diabetes mellitus.	coronary heart disease,liver cirrhosis, left myocardial hypertrophie	SDH, mild SAH, frontobasal hemorrhage, SVD, alcohol-related myopathy of the diaphragma	0	27,27	2	yes	no	no	yes	n.k.	n.k.	no	2	0	0,0039	760	n.k.	n.k.	0	1	72
27	60	m	0	1	0	0	HD, pneumonia	pneumonia,HD	HD	0	36,36	2	no	no	no	no	no	no	no	2	0	0,0319	300	23,9	no	n.k.	n.k.	72
28	59	m	0	0	0	0	sepsis due to pneumokoks with meningitis, status after stroke, status after thrombosis, arterial hypertension, diabetes mellitus	meningitis with focal encephalitis,sepsis due to pneumokoks with multiple organ failure	leptomeningitis with foci of meningoencephalitis, old hemorrhagic lesion in the right thalamus, old infarct in the left putamen	0	18,18	1	yes	yes	yes	yes	yes	no	no	0	0	0,0158	460	n.k.	yes	0	1	48
29	0,0027	m	0	0	0	0	pulmonary hypoplasia, pulmonary hypertension, mekoniumaspiration, pneumothorax, muscular dystrophy type Becker-Kiener, infection	malformation syndrome, respiratory failure	normal brain	0	0,00	0	no	no	no	no	n.k.	no	no	0	0	0,0000	8	n.k.	n.k.	n.k.	n.k.	48
30	69	m	0	1	0	0	ALS, paresis of respiratory muscles	respiratory failure, ALS	ALS, cerebellar microbleeds	0	54,55	2	no	no	no	no	no	no	no	2	0	0,0713	340	19,5	yes	n.k.	n.k.	96
31	52	m	0	1	0	0	status after surgery by diverticulitis of the colon, glomerulonephritis, cognitive impairment, pneumonia, gastrointestinal bleeding, nephrotisc syndrome	multiple organ failure	inactivity related muscular atrophy, no morphological correlate for cogntive impairment	0	45,45	2	no	no	no	no	n.k.	no	no	2	0	0,0110	430	n.k.	n.k.	0	1	48
32	52	f	1	1	0	1	AML, status after bone marrow transplantation, COPD by nikotin abuse, vascular malformations at the liver vessels, status after ASD, posterior revesible enzephalopathy and intracerebral hemorrhage	AML, status after surgery of an ASD	Fresh ICB, posterior reversible enzephalopathy syndrome parieto-occipital, inactivity-related muscular atrophy	0	0,00	2	yes	no	no	no	no	no	no	0	0	0,0000	390	22,3	no	0,5	6	24
33	54	f	0	1	0	0	schizophrenia, SDH, pulmonary embolism?, rhabdomyolysis?	pulmonary embolism	chronic panmyelitis with neurogenic muscular atrophy, SDH, capillary teleangiectasia at the level of the left cingulate gyrus	0	18,18	1	no	no	no	no	yes	no	no	2	0	0,0051	360	34,6	no	n.k.	n.k.	24
34	46	f	0	1	0	0	ALS	ALS	ALS	0	0,00	2	no	no	no	no	no	no	no	0	0	0,0064	205	20,0	no	n.k.	n.k.	120
35	74	f	0	1	0	0	implantation of a hip prothesis, focal epileptic seizures, repiratory failure	atherosclerosis, decompansated left ventricular failure	AGD, SVE	0	90,91	3	no	no	yes	yes	yes	no	no	2	2	0,0213	530	27,3	yes	0	1	24
36	36	m	0	0	0	0	EBV-infektion, sepsis, vena porta thrombosis and liver failure, hemophagic lymphohistiozytosis	liver failure with consecutive multiple organ failure	infarct of the adenohypophysis	0	0,00	0	no	no	no	no	n.k.	no	no	2	0	0,0000	430	n.k.	no	0	1	24
37	53	m	0	1	0	0	coronary heart disease, myocardial infarction, hepatopathy	coronary heart disease, myocardial infarction	Null cell microadenoma of the adenohypophysis	0	18,18	0	no	no	no	no	n.k.	n.k.	yes	2	0	0,0163	500	n.k.	n.k.	0	1	72
38	63	m	1	1	0	1	stenosis of the aorta, IgA-nephropathy, CMV infection	atherosclerosis, heart failure, pulmonary hypertension, liver cirrhosis, renal failure, aortic valve stenosis, failure of the mitral and tricuspidal valves, edema of the lung	lacunar infart left thalamus, fresh microinfarct in CA1, SVD	0	n.d.	2	no	yes	no	no	no	no	no	2	0	0,0703	650	24,0	no	0	1	120
39	46	m	0	1	0	0	right ventricular failure by tricuspidal valve failure	pulmonary hypertension, tricuspidal valve failure, biventricular hypertrophy	normal brain	0	0,00	0	no	no	no	no	n.k.	no	no	0	0	0,0206	485	n.k.	no	0	1	29
40	64	f	0	1	0	0	malignant melanoma	pulmonary embolism, metastasing malignant melanoma	multiple metastases of a malignant melanoma, AGD	0	54,55	2	no	no	no	no	no	no	no	2	0	0,0000	250	28,7	no	0	1	48
41	49	m	0	0	0	0	mitral valve failure, cerebral and peripheral (lower limb) ischemia, ventricular fibrillation	endocarditis of the mitral valve	multiple lacunar infarcts and microinfarcts, large fresh infarct of the right basal ganglia, SVD	0	100,00	2	no	yes	yes	no	no	no	yes	0	0	0,0229	850	23,7	no	0	1	8
42	75	m	2	2	0	1	atrial fibrillation, ICB, infarct of the thalamus, cystic pancreatic lesions, pneumonia	coronary heart disease, pneumonia, chronic pancreatitis, pancreatic carcinoma, diabetes mellitus, renal failure	multiple brain infarcts (thalamus, midbrain, parietal lobe), old microinfarct in CA1, lymphocytic meningoencephalitis	0	36,36	1	no	yes	yes	yes	n.k.	yes	no	2	2	0,0773	425	n.k.	n.k.	0	1	24
43	72	f	4	4	2	2	dementia, arterial hypertension, obesity, lung carcinoma with pleuracarcinosis, atrial fibrillation	coronary heart disease	AD, metastases of a lung adenocarcinoma, SVD, lacunar infarct of the thalamus	1	45,45	2	no	yes	yes	no	yes	yes	no	2	2	0,2221	370	n.k.	n.k.	1	6	12
44	51	m	0	1	0	0	multiple sclerosis,bladder carcinoma, diabetes mellitus, pneumonie	pneumonia	multiple sclerose- Marburg type/ subtype II	0	33,33	0	no	no	no	yes	yes	no	no	2	0	0,0544	400	n.k.	yes	3	7	24
45	45	m	0	0	0	0	myocardial infarction, coronary heart disease	myocardial infarction, coronary heart disease	normal brain	0	0,00	1	no	no	no	no	n.k.	no	yes	2	0	0,0337	410	n.k.	no	0	1	24
46	73	f	2	1	0	1	Obesity, arterial hypertension, renal cell carcinoma, gastric ulcer, decompansated left ventricular failure	decompensated left ventricular failure by status after renal cell carcinoma	ICBs (basal ganglia, thalamus, brain stem cerebellum), SVD	0	66,67	2	yes	no	yes	yes	yes	n.k.	no	2	2	0,1154	725	n.k.	n.k.	0	1	30
47	35	m	0	0	0	0	GIST, endokrine carcinoma of the colon, paraneoplastic encephalitis, factor V Leiden-mutation	GIST, endokrine carcinom of the colon, dilatative cardiomyopathy	limbic encephalitis	0	0,00	0	no	no	no	no	no	no	no	2	0	0,0359	640	27,5	no	0	1	72
48	85	f	0	2	0	0	arterial hypertension, diabetes mellitus, COPD, chronic renal failure, duodenal perforation, sepsis, peumonia	Atherosclerosis, coronary heart disease, old myocardial infarct, decompensated left ventricular failure	SVD, white matter lesions, multiple infarcts (parietal lobe, occipital lobe, hippocampus, thalamus, pons), fresh microinfarct of the adenohypophysis	0	n.d.	3	no	yes	yes	yes	n.k.	n.k.	yes	3	3	0,2252	350	n.k.	n.k.	n.k.	n.k.	48
49	63	m	1	1	0	1	status after atrial ablation, multiple cerebral infarcts	coronoary heart disease, myocardial hypertrophy, fresh myocardial infarct, acute left ventricular failure	multiple white and red infarcts and microinfarcts (cortical ribbon, white matter, thalamus)	0	63,64	2	no	yes	yes	no	no	yes	yes	2	2	0,2208	580	n.k.	no	n.k.	n.k.	48
50	80	m	2	1	0	1	sudden coronary failure	atherosclerosis, aortic valve stenosis, acute heart failure by coronary heart disease	normal brain	0	90,91	2	no	no	yes	no	no	yes	no	2	2	0,3659	510	23,8	no	0	1	48
51	0	m	0	0	0	0	intrauterine death	Intrauterine asphyxia by placental failure	normal fetal brain	0	0,00	0	no	no	no	no	n.k.	no	no	0	0	0,0000	21	n.k.	n.k.	n.k.	n.k.	48
52	76	m	2	2	1	1	atrial fibrillation, heart failure, coronary heart disease, pulmonary emphysema	Atherosclerosis, coronary heart disease, pulmonary emphysema	SVD, CAA	2	36,36	2	no	no	no	no	n.k.	yes	no	2	0	0,5937	640	n.k.	n.k.	n.k.	n.k.	48
53	74	m	1	2	0	1	ALS, coronary heart disease	ALS, respiratory failure	ALS	0	0,00	2	no	no	yes	no	no	no	no	3	3	0,1907	400	19,4	no	n.k.	n.k.	24
54	0	f	0	0	0	0	fetus of 32 gestational week	polycystic kidneys, cardiopulmonal failure	normal fetal brain	0	0,00	0	no	no	no	no	n.k.	n.k.	no	0	0	0,0000	10,4	n.k.	n.k.	n.k.	n.k.	24
55	63	m	0	1	0	0	ALS	lymphoplasmocytic lymphoma	ALS, manifestations of a lymphoplasmozytic lymphom (brain, peripheral nerve, muscle, heart)	0	0,00	2	no	no	yes	no	n.k.	no	no	1	1	0,0110	420	n.k.	n.k.	n.k.	n.k.	48
56	73	m	0	2	0	0	lumbal vertebral bone fracture (L3), status epilepticus, cerebral metastases of a tumor?	non-small cell lung carcinoma, pancreatic carcinoma	metastasis of a non-small cell lung carcinoma, SVD	0	72,73	3	no	no	yes	no	no	no	no	2	2	0,1305	320	n.k.	no	0	1	48
57	74	m	0	0	0	0	COPD, seizures, myocardial infarct	myocardial infarct, adenocarcinoma of the lung	metastasis of an adenocarcinoma of the lung, SVD, infarcts and microinfarcts	0	72,73	2	no	yes	no	no	n.k.	n.k.	no	2	0	0,0420	610	n.k.	n.k.	0	1	72
58	55	m	0	0	0	0	status after ischemia in the territory of the medial cerebral artery, epilepsia	ruptured aneurysm of the infrarenal aorta, atherosclerosis	infarcts (old), aneurysm of the medial cerebral artery	0	36,36	0	no	yes	yes	no	yes	no	no	2	2	0,8039	550	n.k.	yes	n.k.	n.k.	96
59	75	f	4	3	1	2	AML, status after breast carcinoma, renal failure	AML with thrombopenia and hemorrhagic diathesis, SDH	SDH, multiple ICBs by AML, AD, null cell adenoma of the adenohypophysis	1	9,09	1	yes	no	yes	no	yes	no	no	0	0	0,0132	380	29,7	yes	0,5	2	48
60	51	m	0	1	0	0	multiple infarcts, endocarditis, sepsis caused by candida	Signs of sepsis, aortic valve prothesis without signs of infection	ICB in the basal ganglia, multiple infarcts, candida encephalitis associated with sepsis-related bleedings	0	0,00	0	yes	yes	yes	no	yes	no	no	2	2	0,0000	450	30,4	no	0	1	72
61	84	m	3	4	2	2	ALS, dementia, polyneuropathy, chronic alcohol abuse	chronic heart failure, coronary heart disease	ALS, AD, AGD, SVE	2	90,91	3	no	no	no	no	n.k.	no	no	3	0	0,1169	480	n.k.	n.k.	3	7	72
62	57	m	0	1	0	0	infection, coronary heart disease?, hematologic disease?	myocardial infarct, pulmonary emphysema, mitral valve vegetations, atherosclerosis, BPH	normal brain	0	36,36	2	no	no	no	no	n.k.	n.k.	yes	2	0	0,1327	610	n.k.	n.k.	0	1	144

Abbreviations: AβMTL = phase of Aβ-plaque deposition in the medial temporal lobe; AD = Alzheimer's disease; AGD = argyrophilic grain disease; ALS = amyotrphic lateral sclerosis; AML = acute myeloid leukemia; ARDS = adult respiratory distress syndrome; ASD = atrial septum defect; BD = basophilic degeneration of cardiomyocytes; BPH = benign prostate hyperplasia; Braak NFT stage = stage of neurofibrillary tangle pathology; CAA = cerebral amyloid angiopathy; CERAD = consortium to establish a registry for AD; CLL = chronic lymphocytic leukemia; CML = chronic myeloid leukemia; CMV = cytomegaly virus; COPD = chronic obstructive lung disease; EBV = Eppstein Barr virus; f = female; GBS = Guillan-Barré syndrome; GIST = gastrointestinal stroma tumor; GVHD = graft versus host disease; HD = Huntington's disease; ICB = intracerebral bleeding; m = male; NIA-AA = National Institute of Aging - Alzheimer's Association; PD = Parkinson's disease; SAH = subarachnoidal hemorrhage; SDH = subdural hematoma; SVD = cerebral small vessel disease; SVE = subcortical vascular encephalopathy; VD = vascular dementia

Supplementary Tab. 2: Proteins observed for its presence in BD inclusions in cardiomyocytes

	**Cardiomyocyte inclusion bodies**	**Antibodies and staining protocol**
p62/SQSTM1	+	Mouse; Clone 3/p62 LCK ligand, BD Transduction Laboratories, Mountain View, CA, USA, 1:500
Ubiquitin	+	Polyclonal rabbit; Ubiquitin, DAKO, Glostrup, Denmark, 1/100
Ubiquilin	+	Polyclonal rabbit; Anti-UBQLN, Acris Antibodies, San Diego, CA, USA, 1/100, microwave pretreatment
APP-N-terminus (22C11)	+	Mouse; Clone, 22C11, Millipore, 1/75, microwave pretreatment
APP-N-terminus (9023)	(+)	Polyclonal rabbit: 9023, ThermoScientific, Fremont, USA, 1/100, microwave pretreatment
APP-D-Epitope (D-epitope = DALMPSLT)	-	Polyclonal rabbit; 9478D, 9480D, (18), 1/50, microwave pretreatment
APP-M-Epitope (M-epitope = MISEPRISYG)	-	Polyclonal rabbit; 9475M, 9476M, (18), 1/50, microwave pretreatment
$A\beta_{1-17}$ (6E10)	-	Mouse; Clone 6E10, Covance, Dedham, USA, 1/1000, formic acid pretreatment
$A\beta_{17-24}$ (4G8)	-	Mouse; Clone 4G8, Covance, Dedham, USA, 1/5000, formic acid pretreatment
Aβ (3552)	-	Polyclonal rabbit; 3552, (44), 1/1000, formic acid pretreatment
APLP2	-	Polyclonal rabbit: ThermoScientific, Rockford, USA, 1/200, microwave pretreatment
Smooth muscle actin,	-	Mouse; Clone 1A4, DAKO, Glostrup, Denmark, 1/200, microwave pretreatment
Myosin (fast twitch)	-	Mouse; Clone MY-32, Bio-Genex, The Hague, Netherlands, 1/50, microwave pretreatment
abnormal phosphorylated τ-protein	-	Mouse; Clone AT-8, Thermo-Scientific – Pierce Biotechnology, Rockford, IL, USA, 1/1,000
pTDP43 (phosphorylated transactive DNA binding protein)	-	Polyclonal rabbit; pS409/410-2, Cosmo Bio Co., Ltd, Tokyo, Japan, 1/10000, microwave pretreatment
TPD43	-	Mouse; Clone 2E2-D3, Novus Biologicals, Littleton, CO, USA, 1:2000, formic acid and microwave pretreatment
α-synuclein	-	Mouse; Clone KM51, Leica Biosystems – Novocastra, Newcastle, UK, 1/40, formic acid pretreatment
CD-56	-	Mouse; Clone BC56C04, Biocare, Concord, CA, USA, 1/50, microwave pretreatment
Desmin	-	Mouse; Clone D33, Linaris, Dossenheim, Germany, 1/100
Filamin-C	-	Polyclonal rabbit; anti-FLNC, HPA006135, Sigma-Aldrich Chemie Gmbh, Munich, Germany

References:

(18) Willem, M. et al. eta-Secretase processing of APP inhibits neuronal activity in the hippocampus. Nature (2015).

(44) Page, R. M. et al. Beta-amyloid precursor protein mutants respond to gamma-secretase modulators. J Biol Chem 285, 17798-17810 (2010).

Supplementary Fig. 1: Positive controls for immunohistochemistry (**A-D** visualized with avidin-biotin complex and DAB, **E-H** visualized with carbocyanine (Cy) labelled secondary antibodies): **A**: p62/SQSTM1-staining of cytoplasmic dipeptide repeat inclusions in CA4 neurons (arrows) of a case with ALS carrying a C9Orf72 hexanucleotide mutation. The lacking nuclear signal serves as intrinsic negative control. **B, C**: Neuritic plaques (arrows) and less strongly the neuropil and the cytoplasm of neurons of AD brain (**B**: temporal neocortex; **C**: hippocampal sector CA1) are marked with the antibodies against the N-terminus of APP (22C11) (**B**) and the Aη region of APP (D-epitope antibody) (**C**). The nuclei are not stained and serve as intrinsic negative controls. **D**: The anti-$A\beta_{1\text{-}17}$ antibody 6E10 clearly stains amyloid plaques (arrows) in AD temporal neocortex whereas the neighboring neuropil remains negative. **E, F**: Dipeptide inclusions in the granule cell layer of the cerebellum of an ALS case carrying the C9Orf72 hexanucleotide el disease; SVE = subcortical vascular encephalopathy; VD = vascular dementianin-5 (Cy5)-labelled secondary antibodies (**E** and with the ubiquitin antibody-coupled on biotinylated anti-mouse Fab fragments and labelled with Cy2-conjugated streptavidin (**F**). The inclusions stained with both antibodies in this double labelled section are indicated by arrowheads. Ubiquitin antibodies also mark other lesions that are not detected by the p62/SQSTM1 antibody. **G**: The antibody against the M-epitope of the APP-Aη region stains a neuritic plaque with dystrophic neurites (arrowheads) in the parietal cortex of an AD brain (the original Cy 3 color is shown here; the image is not recoded into blue as it is done for triple label immunofluorescence figures!). **H** Amyloid plaque (arrowhead) in the parietal cortex of an AD brain detected with the polyclonal Aβ antibody 3552 visualized with Cy3-labelled secondary antibodies (the original color is shown here; the image is not recoded into blue as it is done for triple label immunofluorescence figures!).

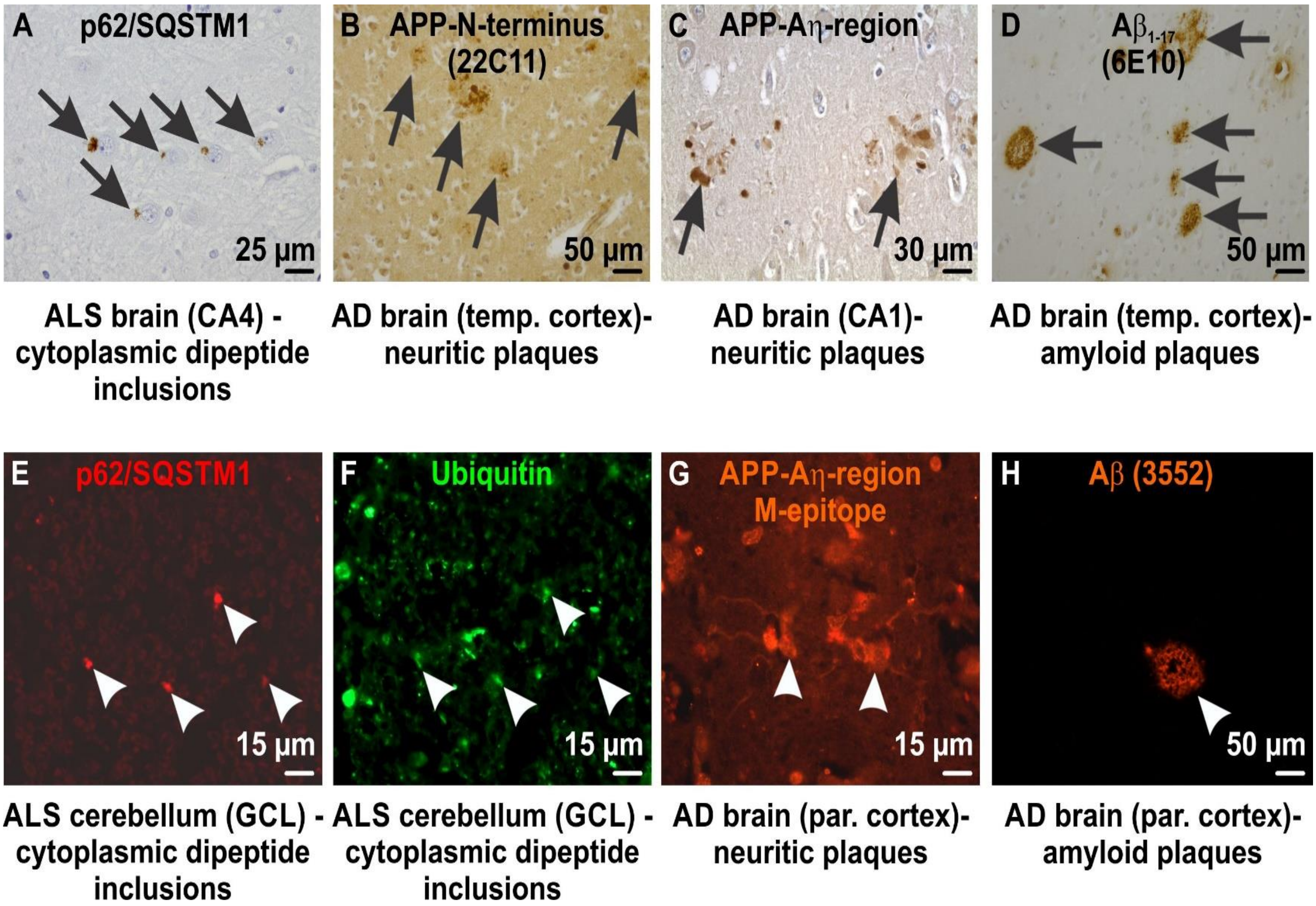

Supplement: Supplementary file 1 — Supplementary Material [file 41598_2018_34808_MOESM1_ESM.pdf]
